# Inno4Vac Workshop Report Part 1: Controlled Human Influenza Virus Infection Model (CHIVIM) Strain Selection and Immune Assays for CHIVIM Studies, November 2021, MHRA, UK

**DOI:** 10.1111/irv.70014

**Published:** 2024-11-04

**Authors:** Joanna Waldock, Rebecca J. Cox, Christopher Chiu, Kanta Subbarao, Adrian Wildfire, Wendy Barclay, Puck B. van Kasteren, John McCauley, Colin A. Russell, Derek Smith, Ryan S. Thwaites, John S. Tregoning, Othmar G. Engelhardt

**Affiliations:** ^1^ Influenza Resource Centre, Vaccines, Science Research & Innovation Medicines and Healthcare Products Regulatory Agency Potters Bar UK; ^2^ Influenza Centre, Department of Clinical Sciences University of Bergen Bergen Norway; ^3^ Department of Infectious Disease Imperial College London London UK; ^4^ WHO Collaborating Centre for Reference and Research on Influenza and Department of Microbiology and Immunology University of Melbourne at the Peter Doherty Institute for Infection and Immunity Melbourne Australia; ^5^ CEO CHIMunomics Surrey UK; ^6^ Centre for Immunology of Infectious Diseases and Vaccines (IIV) National Institute for Public Health and the Environment (RIVM) Bilthoven The Netherlands; ^7^ World‐wide Influenza Centre Francis Crick Institute London UK; ^8^ Amsterdam University Medical Centres University of Amsterdam Amsterdam The Netherlands; ^9^ Centre for Pathogen Evolution, Infectious Diseases Research Centre, Department of Zoology University of Cambridge Cambridge UK; ^10^ National Heart and Lung Institute Imperial College London London UK

**Keywords:** controlled human infection model (CHIM), controlled human influenza virus infection model (CHIVIM), correlates of protection, immune assays, influenza viruses

## Abstract

Controlled human infection models (CHIMs) are a critical tool for the understanding of infectious disease progression, characterising immune responses to infection and rapid assessment of vaccines or drug treatments. There is increasing interest in using CHIMs for vaccine development and an obvious need for widely available and fit‐for‐purpose challenge agents. Inno4Vac is a large European consortium working towards accelerating and de‐risking the development of new vaccines, including the development of CHIMs for influenza, respiratory syncytial virus and *Clostridioides difficile*. This report (in two parts) summarises a workshop held at the MHRA in 2021, focused on how to select CHIM candidate strains of influenza and respiratory syncytial virus (RSV) based on desirable virus characteristics and which immune assays would provide relevant information for assessing pre‐existing and post‐infection immune responses and defining correlates of protection. This manuscript (Part 1) summarises presentations and discussions centred around influenza CHIMs and immune assays (a second manuscript summarises RSV CHIM and immune assays: Inno4Vac workshop report Part 2: RSV CHIM strain selection and immune assays for RSV CHIM studies, November 2021, MHRA, UK).

## 1 Introduction

The development of controlled human infection models (CHIMs) forms part of a large programme of work in the Inno4Vac consortium focused on accelerating and de‐risking the development of new vaccines. Work includes development of open access and cloud based platforms for *in silico* vaccine assessment; new and improved CHIMs for influenza, respiratory syncytial virus (RSV) and *Clostridioides difficile*; cell based human in vitro 3D mucosal models; modular one stop computational platform for *in silico* modelling of vaccine biomanufacturing and stability testing (Inno4Vac project).

A workshop was held at the MHRA to address the requirements of influenza and RSV CHIM development. This workshop focused on influenza/RSV strain selection and immunoassays for CHIM studies. This report summarises the presentations and discussions around controlled human influenza virus infection model (CHIVIM) strain selection and immune assays for CHIVIM studies. A second report summarises RSV CHIM strain selection and immune assays for CHIM studies (Inno4Vac workshop report Part 2: RSV CHIM strain selection and immune assays for RSV CHIM studies).

## 2 Historical Use of CHIMs

The workshop started with a historical overview of CHIM studies using influenza and RSV, highlighting strengths of CHIM studies but also gaps that the consortium aims to fill. The first successful influenza CHIM was in 1936. A series of studies carried out by the Common Cold Research Unit (1946–1989) and elsewhere helped to define the effects of influenza virus strain; dose; route of delivery; transmission and correlates of protection [1]. There are key strengths of human challenge models: defined strain/dose and careful selection of homogeneous study subjects leads to reproducible, and usually high, infection rate; pre‐infection and pre‐symptomatic samples can be taken for assessment along with longitudinal analysis, improving the power to draw inferences with small numbers of participants. Historically, CHIMs have been used in the discovery and validation phases of vaccine development (establishing mechanisms of protection and susceptibility, and discovering correlates of protection using, for example, haemagglutination inhibition (HAI) [1], mucosal antibodies [2, 3], and T‐cell assays [4]). There is, however, increasing interest in further use of CHIM studies in vaccine development, for the rapid early testing of candidates, down‐selection of candidates with low efficacy, de‐risking Phase III trials and contributing to vaccine licensure.

There are limitations of CHIMs: Historically, influenza/RSV CHIMs have produced very mild illness, impacting upon the use of disease endpoints for vaccine development. Additionally, healthy adults were recruited for CHIM studies, who were not fully representative of the vulnerable population requiring protection from disease. These factors will affect the predictive accuracy of any CHIM seeking to predict vaccine efficacy against severe disease, although improved models may help with this.

Existing CHIM strains were discussed (reviewed in [5, 6, 7]) highlighting the need for more contemporary influenza and RSV CHIM strains that causes more representative disease.

## 3 CHIVIMs Within Global Strategy Initiatives

The next speaker focused on the importance of CHIMs within global strategies initiatives. The WHO health research agenda for influenza (Public health research agenda for influenza (who.int)) included the recommendation for human challenge models for preliminary assessment of candidate vaccines. The WHO 2019–2030 Global influenza strategy (Global Influenza Strategy 2019–2030 (who.int)) calls for better global tools to develop novel and universal vaccines, more effective treatments and improved understanding of host–pathogen interactions. CHIMs were discussed as critical to develop better tools centred on testing new antivirals and monoclonal antibody therapies. Recommendations for improving and standardising the methodology of CHIVIMs were given [8]. Recommendations included screening for healthy volunteers (no smokers), including participants with pre‐existing immunity, selecting participants with a range of antibody titres against influenza and balancing those immune profiles within each testing group, to carry out a dose escalation trial and to use nasal atomisers for delivery but avoid aerosol challenge as this is high risk for lower airway infections. More recently, the Centre for Infectious Disease Research and Policy (CIDRAP) published the R and D roadmap for influenza vaccines, including a topic on animal models and CHIVIM (https://ivr.cidrap.umn.edu/roadmap). The roadmap highlights the range of challenge agents required and the need for these to be broadly available. It includes guidance for use cases of CHIVIM, from an ethics and safety perspective. The Inno4Vac project is responsive to many of the recommendations and calls that have come out of recent global strategies for influenza.

## 4 Requirements for Human Influenza Challenge Strains

A perspective of requirements for CHIVIM strain selection was presented, focusing on how to better model clinical disease. Desirable features of a CHIVIM strain include a known route of infection, consistent well‐defined disease and virus characteristics, infectious doses representative of wild‐type (WT) virus exposure, induction of innate and adaptive immune responses, high attack rate (above 60% and preferably 75%), availability of effective intervention treatment and no or preventable long‐term sequelae. Many of these features are present for CHIVIM viruses; however, many are highly strain dependent. Considerations for selecting endpoints were next discussed. Endpoints should reflect the product being tested—for example, treatment studies have used viral load area under the curve (vAUC) (by polymerase chain reaction [PCR]); however, disease incidence (and/or severity) may be more applicable for vaccine development. There is a balance needed between disease severity and required endpoints: The CHIVIM strain should not cause severe disease (e.g., avoiding lower airway infection and the risk of bacterial complications); however, higher rates of fever may be preferable than seen in previous CHIVIM influenza strains. The type of endpoints selected will influence the number of participants required in a CHIVIM study. The use of vAUC (PCR) requires a smaller number of participants for statistical power, whereas the use of symptom scores requires larger numbers due to greater variability of these data. Ideally, good attack rates (proportion of inoculated individual who become infected), good vAUC and measurable symptomology are required.

Dosing considerations were also discussed. High attack rates are desirable; however, it is important that high attack rates are achieved using as representative an infectious dose as possible. Previous studies have shown that breakthrough infection can be induced by giving very high doses of input virus (personal communication, AW), but this may not be representative of natural infection. Increasing doses of virus have been linked to reduced neutralising antibody responses after infection—perhaps indicating overwhelming of the adaptive immune system (personal communication, AW). To model natural disease, good attack rates should therefore be achieved with a dose representative of natural virus infection if possible.

Limiting factors that should be considered include low attack rates (increases participant numbers required), mild symptoms (may increase participant numbers required if using disease readouts), pre‐existing immunity in the population (if high, increases the number of participants for screening to identify those with low strain‐specific immunity, possibly biasing the study group), route of inoculation (may affect infection rates) and inconsistencies in the definition of positive infection that require standardisation (febrile, PCR positive and seroconversion/combination of these).

Identification and isolation of challenge agents were discussed. A preference for swabs from paediatric patients from which virus could be isolated, with clear and uncomplicated medical histories was noted. Collaboration with healthcare institutes was suggested for potential access to viruses during epidemics. Established and predominantly circulating strains offer the greatest relevance for seasonal epidemic disease—newly emergent or pandemic potential viruses may offer unacceptable risk for adverse events or sequelae.

Considerations for virus growth prior to manufacture of CHIVIM agents were discussed. Pilot studies should inform the best growth conditions; testing a number of cell lines and media will enable optimisation of virus growth. Assessment of any adaptive changes is essential during passaging of a challenge agent.

Finally, strategies for retaining WT pathology and viral fitness were discussed. Vaccine strains, mostly amplified in eggs, may already have passage induced attenuation. Inappropriate cell lines or overamplification can lead to truncated proteins [9], changes in glycosylation patterns and accumulation of defective interfering particles (DIPs) [10, 11]. As few rounds of amplification as possible should be carried out. Strategies to preserve viral fitness include selecting a seed stock from a proven virulent strain, assessing mutations after amplification, minimising freeze–thaw cycles, pre‐selecting cell lines most appropriate to viral target and replication cycle, reducing amplification steps, use of multiple suppliers to identify the best media and cell lines for optimal growth and establishing best incubation temperature for growth.

## 5 Existing Genetic and Antigenic Resources for Selection of CHIVIM Candidates

The next two talks focused on the WHO global influenza surveillance and response system (GISRS) network, genetic and antigenic mapping of virus evolution and how data and resources from these activities may be useful for selection and development of CHIVIMs. The GISRS network of 151 National Influenza Centres, 7 WHO Collaborating Centres and 4 Essential Regulatory Laboratories (ERLs) analyses hundreds of thousands of samples each year (Global Influenza Surveillance and Response System (GISRS) (who.int)). The network principally examines genetic sequences of viruses and antigenic differences of viruses [12] (through plaque reduction and HAI assays using ferret sera and serology using human sera) to monitor antigenic drift of virus strains and inform decision making when selecting vaccine virus strains. Both the genetic/antigenic data and possibly the viruses (that are used to develop candidate vaccine viruses [CVVs]) could be used in CHIVIM selection and development. Antigenic cartography has been used to help select candidate CHIVIMs in the past. Viruses that are antigenically distinct from dominantly circulating strains may be less well recognised by immune responses generated by vaccination or natural infection by circulating strains, which in turn may lead to higher attack rates when using an antigenically distinct virus as a challenge agent in a CHIM study. These antigenically distinct clades are often referred to as ‘outlier clades’ or ‘antigenic outgroups’. Examples of the unpredictable nature of influenza virus evolution were highlighted, where a virus from a non‐predominant clade was selected as a CHIVIM candidate; however, in the following influenza season, the selected clade expanded and became the dominant circulating strain in the target population for a CHIVIM study. Thus, there is an inherent risk in selecting contemporary strains from genetic or antigenic ‘outgroups’. Another consideration is that antigenic data for ferret sera may not reflect pre‐existing immunity in humans and the importance of gaining insight into population immunity in the target groups for CHIVIM recruitment was highlighted. This would give useful guidance for likely attack rates of selected CHIVIM candidates.

An alternative to using contemporary virus strains was discussed. Selecting viruses for which the evolutionary history is known, for example, from 30 years ago, genetic and antigenic data could be used to shortlist candidates that would likely have low levels of pre‐existing immunity. Here, a different risk is introduced, where CHIVIM participants would likely have no immunity to an older strain, possibly resulting in more severe infection and raising the issue of epidemic even or pandemic risk.

Further discussion centred on selecting geographically isolated strains that have not circulated globally. These can be contemporary, but because they are isolated, pre‐existing immunity in CHIVIM participants would likely be low. This may come with the caveat of limited clinical information around the isolate, knowledge of which may be useful in CHIVIM development.

The next speaker focused on what drives influenza virus evolution and the impact on human challenge strains. This talk primarily focused on antigenic changes to the haemagglutinin (HA) protein and immune escape, modelling immune responses to infection in order to explain the typical 2–5 year (H3N2) or 3–8 year (H1N1 and B influenza) timescale we see for antigenic variants to spread, when the potential generation of mutant viruses is very fast [12, 13]. Here, the dynamics of infection are important, with the peak of virus shedding occurring before a germinal centre response can develop highly specific antibodies to the infecting virus. The viruses passed on from infected individuals would thus be very similar to the virus this individual was infected with, as the virus has not come under immune pressure yet. The speaker argued that the mucosal IgA response was a barrier to infection and was a likely point of selection of a variant. As mucosal immunity builds up after infection or multiple exposure to antigenically similar viruses, this will become a bottleneck, only allowing antigenic variants to subsequently infect an individual [13]. Screening individuals for recruitment into a CHIVIM for mucosal IgA titres may be very useful for identifying susceptible individuals.

## 6 In Vitro and In Vivo Models of Influenza to Guide Selection of Challenge Strains

The next speaker gave an overview of in vitro and in vivo models to guide selection of challenge strains for next generation influenza vaccines. A challenge strain should represent a typical human influenza virus, be competent for replication in the human upper airway tract, with airborne transmission and unlikely to cause lower airway infection. Efficient replication in the upper airway requires an HA that binds long‐chain alpha2‐6 sialic acid (SA) receptors [14], an HA stable to mildly acidic respiratory secretions [15], a balanced neuraminidase (NA)/HA affinity for SA receptors [16], the ability to overcome innate immunity and a polymerase that works efficiently at 33°C [17].

Considering in vitro models, primary human airway epithelial (HAE) cultures from human nasal passages or trachea‐bronchial airways were cited as a good model for identifying viruses fit to grow in upper airways. Primary HAEs have ciliated epithelial cells, goblet cells and an airway interface. The HAE model closely represents morphological and physiological features of the human airway [18] supporting growth of human but restricting avian influenza viruses [18, 19]. These cultures reflect some of the barriers to virus infection and growth. HAEs could be useful for conducting fitness studies of candidate CHIVIMs. In vivo models were discussed, with agreement that the ferret model is the best available option. Mice models have different sialic acid receptors for influenza virus infection to humans and Guinea pig and hamster models are under‐used and poorly characterised. Ferrets show clinical signs during influenza infection [20, 21] and shed virus that is transmissible by air [22]. A combination of in vitro characterisation in HAEs as an indicator of viral fitness and in vivo characterisation in ferrets was recommended. Inno4Vac partners have access to both these models, and assessment of CHIVIM candidate viruses is included in the pipeline of CHIVIM development.

## 7 CHIVIMs as a Tool for Influenza Vaccine Development

The final talk of this session discussed what type of strains of influenza we need for CHIVIMs for vaccine development. For the purposes of the talk, influenza vaccines were divided into ‘improved seasonal vaccines’ that are like existing vaccines, ‘broadly protective vaccines’ that protect within a subtype, and ‘universal vaccines’ that protect across subtypes.

Benefits of a CHIVIM were discussed in the context of each category of influenza vaccine. It was argued that CHIVIMs would not be beneficial for the development of improved seasonal vaccines: HAI titres (the main, and most robust, readout for seasonal influenza vaccines [23]) already de‐risk projects; immunogenicity‐based Phase III trials are potentially cheaper than large scale CHIVIM trials; and improved seasonal vaccines are aimed at ‘at‐risk’ groups (elderly, immunocompromised) who are not included in CHIVIM trials. However, CHIVIMs would be beneficial for broadly protective and universal vaccine development, as breadth of protection can be explored using challenge models. For broadly protective vaccines, CHIVIMs could use seasonal viruses that are sufficiently antigenically distinct to circulating viruses avoiding issues of pre‐existing immunity. Historical viruses could be used, but the biosafety implications of this must be considered (including high containment requirements and risk to the public of virus escape). For universal vaccines, CHIVIMs would be very useful for de‐risking trials and for claims of universality [8]. In this case, strains of interest would include H2N2, H6NX, H5NX, H7Nx, H9N2 and H10NX subtypes; however, the use of such viruses as challenge agents would likely represent an unacceptable risk of pandemic potential for ethics approval, without the existence of a rescue therapy or a ‘suicide mechanism’ to prevent viral escape. There may be challenges with using some of these viruses as CHIVIMs, for example, WT avian viruses replicate very poorly in humans [24]. A further caveat of using CHIVIM studies to show breadth of protection stems from whether or not regulators will accept CHIVIM studies in their framework of decision making [8], highlighting the importance of involving regulators in projects such as Inno4Vac to maximise the usability of CHIVIMs in vaccine and drug development.

The discussion sessions centred around several main questions: which influenza strains would be most suitable for CHIVIM and what steps should be taken to ensure strains meet the criteria for a successful CHIVIM. Table 1 summarises the advantages and disadvantages to be considered for selection of virus strains for CHIVIM development.

**TABLE 1:** Advantages and disadvantages of influenza strains that could be selected as CHIVIMs.

Advantages				
Older virus (20–30 years)	Antigenic outlier virus	Contemporary virus	Reverse genetic virus	Zoonotic virus
Likely low pre‐existing immunity	Antigenically distinct from dominantly circulating strains—can be used to probe broader protection of vaccines within a subtype	Likely low pre‐existing immunity (if not widely circulating)	No adventitious agents	Could be used to show breadth of protection of universal/broadly protective vaccines
	Immunological history known—immune profiles unlikely to change over time	Best represents circulating strains		
	Likely lower levels of pre‐existing immunity than to circulating strains	Original isolate and clinical information likely to be available		

Disadvantages				
Older virus (20–30 years)	Antigenic outlier virus	Contemporary virus	Reverse genetic virus	Zoonotic virus
Probably isolated in eggs—likely egg adaptation that may attenuate growth	May be outliers for a biological reason—for example, poor growth	Limited lifespan of the CHIVIM as immunity in the population increases—would need replacing every 5 years or so	More manipulation—may affect the virology compared to WT viruses	Safety concerns—use of zoonotic viruses represents a pandemic potential risk
Large global population with little to no immunity—safety issues with possible escape into the community	Could return as a dominant clade (depending on how contemporary the outlier is)		Previous experience suggests RG viruses have attenuated pathology	Unlikely to get regulatory approval to use zoonotic challenge strains
Limits CHIVIM participants to younger age groups—clinical bias			RG viruses may encounter issues with regulatory approval in certain countries	Avian viruses often have poor infectivity in human hosts
May not have access to original swabs or clinical information				

Abbreviations: CHIVIM = controlled human influenza virus infection model, WT = wild type.

A number of recommendations broadly split into three categories were made:

*Initial characterisation of a strain/panel of strains*

- In vitro characterisation of growth would be important to mitigate any issues of antigenic outliers having poor growth characteristics.
- In vitro studies using HAEs would be beneficial for identifying likely fitness of strains during human infection.
- In vivo characterisation using a ferret model would provide useful symptomology and virological data. Lower airway infections and severe pathology should be avoided.

*Serological characterisation*

- Assessing the level of background immunity to strain of interest using panels of human sera from target populations would provide useful information. HAI and MN assays would be appropriate.

*Growth/amplification of the virus*

- Strains isolated and passaged in suitable cell lines are preferable to those isolated and passaged in eggs. Viruses passaged in eggs usually acquire egg adaptation mutations, and these may reduce virus fitness in the human host.
- Avoid using any process that creates a virus that is less fit for replication in humans—this leads to higher doses being required for challenge trials, which is not representative of real human infection and disease.
- Cell line must be well characterised.
- Growth media, temperature and growing conditions are very important—pilot studies would inform optimisation of culture conditions

A protocol for comparison of CHIVIM strains was developed based on the discussion in this workshop (see Supporting Information, ‘Protocol for comparison of CHIVIM strains’). This document gives guidance on a recommended procedure for identification and assessment of CHIVIM candidate strains.

## 8 Immune Assays for Use in CHIVIM

### 8.1 Importance of Immunity for Influenza Infection

The first talk focused on the importance of immunity to influenza infection. The immunological history of an individual will influence their susceptibility to infection. A key question is how to select immune assays for measuring both pre‐existing immunity to infection prior to a CHIVIM study and for measuring immune responses to infection during a CHIVIM study. Current regulatory criteria for licensure of new seasonal or pandemic influenza vaccines requires vaccine manufacturers to conduct trials examining tolerability and immunogenicity [25]. The HAI and single radial haemolysis (SRH) assays are currently used for measuring immune responses to vaccination, and both have been shown to correlate with protection against infection. Multiple studies indicate that antibody responses measured with HAI are a surrogate correlate of protection [1, 23, 26]. However, the variety of vaccine types in use and in development (live attenuated influenza vaccines [LAIV], inactivated virus, mRNA and recombinant protein) necessitates assays for measuring alternative correlates of protection. The LAIV serves as an example; here, serum HAI titres have been shown to be very low after LAIV vaccination despite good vaccine efficacy [27, 28]. For LAIV, HAI is a poor correlate of protection, and other immune assays may provide a better indicator of protection induced by vaccination. High levels of anti‐HA stalk antibodies have been shown to correlate with reduced viral shedding but are not a predictor of disease severity [29]. Anti‐neuraminidase antibodies measured using neuraminidase inhibition assays (NAI) have been shown to be stronger predictors of disease outcome and severity than HAI in several studies [29, 30, 31], nasal IgA can protect against influenza infection [32], and T‐cell immunity has also been shown to be important in protection against shedding and infection outcome of influenza [33, 34]. Within this consortium, there is an opportunity to fully characterise pre‐existing immunity, considering the multifaceted responses to infection, and help define host susceptibility to infection.

### 8.2 Standardisation of Influenza Immune Assays

The next talk a summarised of the standardisation activities that took place during the FLUCOP project (FLUCOP – Standardisation and development of assays for assessment of influenza vaccine correlates of protection). FLUCOP was an IMI‐funded consortium that aimed to develop standardised assays for assessing influenza correlates of protection. The primary goals to standardise the HAI and microneutralisation (MN) assays were discussed, where several large‐scale collaborative studies were carried out to develop and assess harmonised protocols and test biological standards, with publicly available protocols now published for HAI [35, 36] (along with a training video FLUCOP Training module (figshare.com)) and microneutralisation assays [35]. Further goals of the FLUCOP project were to validate an enzyme‐linked lectin assay (ELLA) for measuring NAI titres [37] and to standardise PBMC isolation and T‐cell immune response assays [38, 39, 40]. It is strongly recommended that these harmonised and tested protocols are adopted by laboratories to help reduce levels of interlaboratory variability in current HAI, MN and ELLA testing. Much of the data generated during the FLUCOP project has been published in a special issue in Frontiers in Immunology on ‘Development and standardization of assays to assess immunogenicity and correlates of protection of vaccines against respiratory viral infections’ (Development and standardisation of assays to assess immunogenicity and correlates of protection of vaccines against respiratory viral infections | Frontiers Research Topic (frontiersin.org)).

The next talk focused on developing an antibody standards for HA stalk antibodies. Antibodies directed against the stalk region of the influenza HA protein are known to be cross reactive within and between subtypes. Some of the next‐generation influenza vaccines target the stalk of HA. Assays currently used to measure anti‐stalk antibodies are either functional (neutralisation assays and antibody‐dependent cellular cytotoxicity [ADCC]) or binding assays (ELISA). Serological readouts from vaccine trials of HA stalk‐targeting vaccines may serve as benchmarks or CoPs, and as such, standardised assays and/or biological standards are desirable for comparison of clinical trial data and potential definition of a CoP. A project to develop an antibody standard used a pool of serum samples with high titres of anti‐stalk antibodies, characterised and tested as a standard across multiple laboratories [41]. This was shown to reduce interlaboratory variation in binding assays; however insufficient data were obtained for neutralisation assays. A second phase of the project will aim to create a WHO International Standard (IS).

### 8.3 Correlates of Protection and Selection of Appropriate Immunological Measurements

A summary of a challenge study using LAIV in healthy young adults in 2018–2019 was discussed, where differences between local and peripheral antibody responses were investigated [42]. Correlates of protection for LAIV are not well understood—in children, efficacy is higher than traditional correlates suggest [27, 28]. One purpose of the study [42] was to try and identify what was driving efficacy, focusing on mucosal humoral responses to LAIV between baseline samples and 28 days post infection. By measuring nasal antibody responses (using nasal sampling methods [43]) and peripheral antibodies, it was found that some individuals responded both locally and peripherally, some responded only locally and some only peripherally. Nasal and serum responses were largely not correlated with each other. This indicated that the mucosal response may be at least partially independent of systemic humoral immune responses. Further evidence from PBMCs showed distinct immune responses between those participants that formed either systemic IgG or nasal IgA responses. Participants who had an activated pool of memory cells (antibody‐secreting cells [ASC] and circulating T follicular helper cells [cTfh] activation) showed an inverse correlation with serum IgG responses—participants who did not activate a pool of memory cells had a new serum IgG response and seroconverted, those with activated ASC/cTfh cells did not induce a new IgG response in the periphery. Pro‐inflammatory responses were correlated with mucosal antibody responses. There appear to be distinct immunological events happening in participants with peripheral antibody sero‐conversions versus mucosal antibody sero‐conversion after LAIV infection. For LAIV and vaccine studies, identifying mucosal antibodies is important as HAI will not capture these distinct mucosal responses.

The next talk was on the role of local IgA in influenza vaccination and infection. Correlates of protection are individual, with layers of immune components that will offer protection—an individual may have high a mucosal IgA response that confers protection, or an individual may have a robust IgG systemic response that confers protection. Ideally, we would have multiple measurements to capture these different responses and define a CoP. IgA levels are associated with susceptibility to infection: Several challenge studies have linked low levels of IgA with susceptibility to infection or symptomatic infection [32, 44, 45, 46, 47, 48]. There is limited evidence that the same is true for RSV infection [2, 49]. LAIV studies show weak evidence that high levels of baseline IgA may limit LAIV infection/and or virus shedding for some influenza strains [32, 50], but no link was observed in the Flushed study (Flushed: nasal flu vaccine study (publishing.service.gov.uk)) between baseline IgA levels and fold changes in systemic IgG responses post vaccination [51].

It is important to think about which layers of protection are useful for pre‐screening of volunteers or which are of interest for measurement during challenge studies. Standardised methods for sampling of the mucosa are additionally important—different techniques may be measuring different compartments of the upper respiratory tract (e.g., synthetic absorptive matrix [SAM] strip sampling vs. nasal washes). This should be considered for any challenge study.

The Inno4Vac project offers an opportunity to try to combine mathematically multiple measurements to generate a correlate of susceptibility ‘score’ that would incorporate baseline quantity and quality of local and systemic IgG, IgA and T‐cell responses.

### 8.4 In Vitro Respiratory Models

A summary of the in vitro respiratory models to be utilised by Inno4Vac was presented. The development of novel and improved respiratory virus vaccines can benefit from the use of complex in vitro respiratory model systems, as these may provide important insights into mechanisms of protection and disease and will reduce the need for small animal models that are often poorly representing the human situation. The ultimate aim of the Inno4vac respiratory models work package is to develop novel in vitro models that include immunological components and can be used for vaccine research.

This work package encompasses different model systems that are together suitable to address a variety of vaccine‐related research questions. Initially, three infection model types will be developed including air–liquid interface nasal and bronchial epithelial transwell models, an air–liquid interface alveolar lung‐on‐chip model and bronchial and alveolar organoid models, which will be assessed for influenza virus and RSV infection in preparation of the future addition of immune components. Virus strains that will be tested are required to grow to sufficient titres, be grown on cells not eggs (influenza) and be wild‐type virus (not recombinant/vaccine). Where possible, strains tested will be aligned with strains being selected as CHIM candidates within the Inno4Vac consortium.

## 9 Conclusions and Closing Remarks

This workshop brought together a wide range of experts on respiratory viruses, specifically influenza, providing a wealth of information on desirable CHIVIM strain characteristics and the tools available to guide selection and pre‐manufacture assessment of candidate CHIVIM strains. These discussions paved the way for developing a protocol for CHIVIM development, included in the supplementary materials of this manuscript.

## Author Contributions

Conceptualisation: R.J.C., C.C. and O.G.E. Presentations/discussions: J.W., R.J.C., C.C., K.S., A.W., W.B., P.B.v.K., J.M., C.A.R., D.S., R.S.T., J.S.T. and O.G.E. Writing manuscript: J.W. Reviewing and editing manuscript: J.W., R.J.C., C.C., K.S., A.W., W.B., P.B.v.K., J.M., C.A.R., D.S., R.S.T., J.S.T. and O.G.E.

## Conflicts of Interest

J.W., R.J.C., C.C., K.S., W.B., P.B.v.K., J.M., C.A.R., D.S., R.S.T., J.S.T. and O.G.E. declare no conflicts of interest. AW is the CEO of CHIMunomics.

### Peer Review

The peer review history for this article is available at https://www.webofscience.com/api/gateway/wos/peer‐review/10.1111/irv.70014.

## Supporting information


**Data S1** Supporting Information.

## Data Availability

No new data was generated in this study.
