# Supplementary material for: Inno4Vac Workshop Report Part 1: Controlled Human Influenza Virus Infection Model (CHIVIM) Strain Selection and Immune Assays for CHIVIM Studies, November 2021, MHRA, UK
Source: Influenza Other Respir Viruses. 2024 Nov 4;18(11):e70014. doi: 10.1111/irv.70014 (PMC11534430; doi:10.1111/irv.70014)
Supplement: Supplementary file 1 — Data S1 Supporting Information. [file IRV-18-e70014-s001.docx]

**Inno4Vac workshop report Part 1: Controlled human influenza virus infection model (CHIVIM) strain selection, November 2021, MHRA, UK.**

# **Supplementary material**

# **Protocol for comparison of CHIVIM candidates**

## Introduction

The development of Controlled Human Infection Models (CHIMs) forms part of a large programme of work in the Inno4Vac consortium focused on accelerating and de-risking the development of new vaccines. The breadth of work of Inno4Vac includes: development of an open access and cloud based platform for *in silico* vaccine assessment; new and improved CHIMs for influenza, RSV and Clostridium difficile; cell based human *in vitro* 3D mucosal models to better mimic the *in vivo* immune response; modular one stop computational platform for *in silico* modelling of vaccine biomanufacturing and stability testing (ref inno4vac website).

Two workshops were held (October 2021 and January 2022) at the MHRA to specifically address the requirements of influenza and RSV CHIM development. The first focused on influenza/RSV strain selection (historical aspects and required features of CHIM virus strains) and immunoassays. The second focused on influenza/RSV strain selection and regulatory frameworks for CHIM studies. One outcome of these workshops after discussion of the selection criteria for Controlled Human Influenza Virus Infection Models (CHIVIMs) was to develop a pipeline for selection of CHIVIM candidates using a staged approach to assess strain suitability using genetic, antigenic and growth characteristics. This document gives a summary of the selection criteria and CHIVIM development process utilised by the Inno4Vac consortium.

**Summary of Pipeline for selection of candidates**

1. Strains with low levels of existing immunity in the target population (18–55-year-old adults) will be selected based on available genetic/antigenic characterisation and on one out of three possible strategies:
   1. Antigenic outgroups within the last 10 years. Strains that were:
      1. geographically isolated and did not spread globally or
      2. were replaced rapidly by other antigenic groups, were not dominant strains and thus pre-existing immunity in the general population should be relatively low.
   2. Older strains, to which clinical trial participants would have little pre-existing immunity; this must be balanced with the risk of re-introduction of a virus, leading to outbreaks (epidemic and pseudo-pandemic).
   3. Recent strains (with an assumed relative short lifespan as a CHIVIM requiring replacement every 5 years or so).
2. Virus isolation
   1. Isolation methods.
   2. Best practise storage of isolates.
   3. Sequencing.
   4. Documentation around clinical data available for the isolate, and consent for use.
3. Initial growth of selected CHIVIM strain stock for further characterisation
   1. Want to achieve an HA titre greater than 1:4, so the virus can be used in HAI.
   2. Prefer strains that grow well – important for infectious dose. Historically influenza virus has been administered in CHIM studies at 3-7.2 log_10_ TCID50 per dose[1].
   3. Viruses should be sequenced before and after any amplification step and amplification steps should be kept to a minimum. Low passage strains are preferable.
   4. Viruses should preferentially be grown in mammalian cells to minimise changes in WT viruses and adaptation to growth in eggs.
4. Serologic characterisation of strains
   1. Identify and source suitable human serum panel/s for population serology testing – preferably from the target population for an influenza CHIM trial (e.g. pre-screening samples from existing clinical trials). Sera should be contemporary if possible (e.g. collected after the most recent influenza season to better reflect current population immunity to influenza strains).
   2. HAI using contemporary target population sera. A consensus protocol for HAI can be found in [2].
   3. MN using contemporary target population sera. A consensus protocol for MN testing can be found in [3].
5. Selection criteria based on serology results
   1. % HAI seronegative – typical admission into a clinical trial for influenza infection requires HAI to be seronegative for the CHIM strain. % HAI seronegative levels of at least 50% is desirable (to reduce the number of participants that require screening for entry to a study)
   2. % MN titre <20 – typical admission to a clinical trial for influenza infection requires an MN titre of <20 for the CHIM strain. % MN titres <20 of at least 50% is desirable (to reduce the number of participants that require screening to entry to a study).
6. *In vitro* characterisation
   1. Selection of appropriate cell lines – MDCK, MDCK-SIAT, A549, VERO
   2. Growth of strains assessed by TCID50 and plaque assay
   3. Genetic sequencing of HA and NA during passage to assess genetic stability
   4. Growth in Human Airway Epithelial cells (HAEs) and possibly relevant 3D cultures
7. *In vivo* characterisation
   1. Ferret model to be used for *in vivo* characterisation
      1. Symptomology
      2. Virological data
8. Review of *in vitro* and *in vivo* data to select final CHIVIM candidate/s
   1. Review and discussion of genetic, antigenic, *in vitro* and *in vivo* data to select CHIVIM strain for production.

# **Pipeline for selection of candidates**

## Selection of strains using existing genetic characterisation

A major hurdle that must be overcome for a CHIVIM strain to be used within a clinical trial is the pre-existing immunity that all individuals have to influenza viruses based on their complex immunological history of infection. Selection of a CHIVIM strain should include the use of existing genetic and antigenic data to identify influenza viruses that are likely to be less well recognised by the immune systems of target populations (in this case 18-55 year olds). Several strategies could be used to achieve this:

1. H1/H3/B Antigenic outliers – find a relatively recent (but not contemporary, so the evolutionary history can be seen) strain/s that is an antigenic outlier to select a candidate. Antigenic outliers are strains that have had limited geographical spread or have rapidly been replaced by other clades in recent history. These strains have not circulated globally and pre-existing immunity should be lower than immunity to the dominantly circulating strains. A suggested time window of 10 years would limit pandemic potential of such strains.
2. Older H1/H3/B viruses – go back in time (20-30 years or use a more nuanced approach looking at epitopes to select an appropriate time-period) so that participants are likely to have little immunity – there is a balance of risk here considering epidemic and pandemic potential if the CHIVIM escapes containment. There may be problems with ethical approvals for such virus strains in the absence of an engineered mechanism for control of the virus (such as a suicide switch) or rescue therapy.
3. Contemporary H1/H3/B, with a view to these CHIVIM strains needing to be updated every 5 years or so, with low seropositivity in the target population – this assumes that pandemic/zoonotic viruses are out of the scope of this project.

All three strategies assume that pandemic/zoonotic viruses are out of scope.

The GISRS network collects and sequences viruses from across the globe. Phylogenetic trees can provide useful information on the geographical distribution of influenza strains and can be used to identify clades of viruses that have been replaced in global circulation. Nextstrain.org is a useful tool for visualisation and selection of specific clades and strains of influenza viruses of interest.

The Inno4Vac consortium agreed upon an initial selection of several clades for further characterisation, including geographically isolated clades, clades that circulated previously but were replaced globally within a restricted period of 10 years, and some contemporary strains currently circulating. These shortlisted clades would be tested for levels of pre-existing immunity within the target population.

## Isolation of virus strains

Virus isolates should be stored in multiple aliquots to avoid multiple freeze-thaw cycles. The original swab should be retained for the possibility of re-isolation. Isolated virus should be stored at or below -70C. Methods for virus isolation can be found in Peck et al 2021 [4].

Ideally, well characterised clinical information about the isolates should be documented, along with the appropriate consent for the isolate to be used in CHIVIM development. Isolated viruses and any subsequent passages should be sequenced to identify any genetic changes.

## Initial growth of CHIVIM strains for further characterisation

After identification of genetic clades of interest and virus isolation, CHIVIM strains must be propagated prior to serology screening to assess background levels of pre-existing immunity. Influenza viruses can be propagated either in embryonated chicken eggs using standard protocols or can be propagated in cell culture. To avoid adaptation to avian hosts and minimise changes to the WT virus, amplification in mammalian cell culture is recommended. A stock of the lowest passage of any strain should be retained – this ensures low passage virus can be used to generate a challenge stock if selected as a CHIVIM strain.

Different influenza subtypes require specific culturing conditions for optimal growth. H1N1 and B viruses are typically propagated in MDCK cells. H3N2 viruses are typically propagated in MDCK-SIAT cells (MDCKs that overexpress alpha 2,6-linked sialic acids and overall support improved influenza virus isolation and growth [5]). Growth media and culture condition recommendations can be found from cell suppliers.

Virus growth should be assessed by HA titre (using either turkey Red Blood Cells (RBCs) for H1N1 and B viruses, or guinea pig RBCs for H3N2 strains) and by standard TCID50 or plaque assay methods. A minimum HA titre of 1:4 is required so that assessment of antibody recognition using Haemagglutination Inhibition (HAI) assay is possible. H3N2 strains from 2005 onwards often show greatly reduced ability to agglutinate RBCs [6] and HA titres may be very low despite good virus growth. For these viruses, assessment of antibody recognition is preferentially carried out using a microneutralisation assay, which negates the need for RBC agglutination.

Historically influenza strains in CHIM studies have been administered to participants at 3-7.2 log_10_ TCID50 per dose [1]. Considering this the TCID50 of CHIVIM candidates should be in the range of 5-7 log10 TCID50.

Viruses should be sequenced (a minimum of the HA and NA sequences) before and after any amplification step to assess genetic stability of the CHIVIM candidate.

If appropriate ferret antisera are available, it is recommended that amplified viruses are compared antigenically to their parent virus and/or reference viruses representing the clade chosen, using HAI and/or VN assays.

## Serologic characterisation of CHIVIM strains

After initial selection of genetic clades and amplification of a shortlist of strains in mammalian cell culture, serological screening of virus strains to estimate background levels of pre-existing immunity is recommended.

Firstly, suitable serum panel/s must be identified for testing. These should represent the target population of the CHIM studies for which the strain will be used. Broadly this will be healthy adults within the age range of 18-55, preferably from the country/region in which CHIM studies will be carried out, as there will be considerable global variation due to differences in virus exposure history. The Inno4Vac consortium has sourced screening samples from existing clinical trials. Testing sera should ideally be collected after the most recent influenza season, as this will best reflect current background immunity in the population. This is particularly important if testing recently circulating influenza strains.

Screening for background immunity using two serological methods is recommended:

HAI – The HAI assay quantifies antibodies that bind to the globular head of the HA protein, disrupting the interaction of HA and sialic acid residues on RBCs and inhibiting the cross linking of red blood cells by the virus. Serially diluting sera allows for quantification of antibodies that inhibit red blood cell agglutination. A comprehensive SOP for HAI testing was developed and standardised by the FLUCOP consortium and is available with open access [2, 3].

MN – the Microneutralisation assay quantifies antibodies that prevent infection of cells by binding to and neutralising virions. This assay measures functional antibodies and may be more biologically relevant for assessing susceptibility to infection than HAI. Additionally, this assay can be used to assess immunity to H3N2 viruses that have lost the ability to agglutinate RBCs, and thus cannot be assessed using HAI. An MN protocol has been developed and tested by the FLUCOP consortium and is available with open access [3].

If reagents are available, additional screening of anti-neuraminidase antibodies may be of interest. A fully validated SOP is available with open access [7].

## Shortlist candidates for further characterisation

Historically an HAI titre of 1:40 has been shown to give 50% protection against influenza infection [8]. If participants for a CHIM study are not pre-screened, we would expect a proportion of them to have high enough pre-existing immunity to protect them from infection. Participants are therefore screened for pre-existing HAI (or MN) titres and those with high levels of antibodies are excluded i.e. only sero-suitable participants are accepted. A CHIVIM strain with low pre-existing immunity will reduce the number of participants required for pre-screening, will increases the chances of viable recruitment into a CHIM study, and ensure a sufficient attack rate is achieved within the study.

A review of historical influenza CHIMs showed that overall ~88% of participants with an HAI<1:16 were susceptible to infection [1]. Seronegativity (or an HAI titre<1:10) is often used as a criterion for sero-suitable acceptance into an influenza CHIM. Serology screening using HAI can be used to estimate the likely percentage of sero-suitable individuals. CHIVIM candidates ideally will have a high percentage of sero-suitable titres during population immunity screens.

MN assays are known to be more sensitive than HAI at detecting low levels of antibodies [6, 9-11]. A commonly used criterion for sero-suitability pre-screening using MN is a titre of <1:20, however it should be noted that there is currently no defined correlate of protection for MN assays. Again, CHIVIM candidates ideally will have a high percentage of MN titres <1:20 in population immunity screens.

HAI assays are quick, inexpensive, require little in the way of specialist equipment and have a defined correlate of protection, making this assay the preferred option for CHIVIM study strain pre-screening.

## *In vitro* characterisation

Genetic and serologic/antigenic screening can be used to identify strains of interest. Subsequently identified CHIVIM candidate strains should be subject to *in vitro* characterisation to optimise growth and assess genetic stability.

One of the primary goals of an influenza CHIM study is to model natural influenza disease. A challenge strain should be able to replicate efficiently in the human upper respiratory tract, binding alpha 2,6-linked sialic acids. Steps should be taken to avoid adaptation to culturing conditions and loss of infectivity, thus maintaining WT strain characteristics:

1. Amplification of virus stocks in mammalian cell culture is preferred avoiding common egg adaptations [4].
2. Minimal amplification cycles should be carried out to reduce the risk of virus adaptation to cell culture conditions, and accumulation of defective interfering particles (DIPs). Additionally, a low MOI should be used during amplification and infectious titres should be measured. DIPs have been shown to correlate with severity of disease outcome [12] – it is possible that the typically mild symptomology seen in historical CHIMs may be due in part to accumulated DIPs in over amplified challenge strains. Recent advances in NGS allow quantification of DIP formation during cell passage [12].
3. Sequencing should additionally be carried out at each amplification stage to assess genetic stability of the isolate.

Cell lines that support influenza growth include Vero, MDCK, MDCK-SIAT, A549 and 293T although MDCK cells are most commonly used. Testing growth media from multiple suppliers is recommended to optimise growth. A series of pilot studies should inform the optimal cell line and growth media for virus amplification with minimal changes to the WT virus sequence. Growth media should be suitable for GMP production.

Infectious titres should be measured using standard TCID50 or plaque assay techniques. Quantitative real time PCR can also be used to assess viral load, however this is not a measurement of infectious viral particles and may be affected by the presence of DIPs.

In addition to growth kinetics in standard cell culture, the use of primary Human Airway Epithelial (HAE) cells or other respiratory organoid culture models is also recommended as an *in vitro* model of human infection. Primary HAEs have ciliated epithelial cells, goblet cells and an airway interface. The HAEs model closely represents the morphological and physiological features of the human airway [13] supporting growth of human but restricting avian influenza viruses [13, 14]. HAEs are recommended as an indication of fitness for CHIVIM candidates.

## *In vivo* characterisation

Multiple animal models exist for influenza infection, however the ferret model is considered the most representative of human infection. *In vivo* characterisation of a final shortlist of strains in ferrets is recommended. Of interest are:

- - 1. Symptomology
       1. body temperature
       2. body weight
       3. behaviour
       4. monitor for other signs of disease, for instance neurological symptoms, lack of appetite, sneezing, nasal discharge
    2. Virological data
       1. virus titre in nasal washes
       2. virus titre in lungs (post-euthanasia)
       3. virus titre in nasal turbinates (post-euthanasia)
       4. serum antibodies (2 weeks p.i.) by HAI and VN

Ferrets must be tested for anti-influenza antibodies prior to use in studies to confirm they are naïve for influenza infection. Symptomology and virological data from the ferret model will give an indication of viral fitness in the human host. There are specific criteria by which a candidate strain would be excluded from selection as a CHIVIM – for example mortality, severe pathology in the lower airways etc. If severe pathology is observed, further characterisation of *in vivo* infection can be carried out (e.g. virus in olfactory bulb, virus titre in other organs if systemic infection is suspected).

## Review and final selection of CHIVIM strain

Final review of the data to assess strain suitability prioritising the following characteristics:

1. Sero-suitable immunity in the target population – high percentage of HAI seronegative individuals in the target population (or HAI<1:10) with respect to the selected strain is desirable, high percentage of MN<1:20 is desirable.
2. Good titre when amplified in optimised cell line – 5-7 log_10_ TCID50 recommended minimum, HA titre 1:4 minimum.
3. Restricted rounds of amplification in mammalian cell culture only – as few passages as possible to retain WT pathology and avoid adaptation.
4. Genetic stability during amplification demonstrated.
5. Able to infect and replicate in HAEs.
6. Induction of symptoms (nasal discharge, sneezing, elevated body temperature, possibly reduction of body weight) in ferrets and replication of virus in upper respiratory tract, with possible replication also in lower respiratory tract. Absence of mortality or severe pathology.

Once a candidate strain has been selected, either the original clinical material or the earliest passage possible should be used for production of the CHIVIM.

## Bibliography

1. Carrat, F., et al., *Time lines of infection and disease in human influenza: a review of volunteer challenge studies.* Am J Epidemiol, 2008. **167**(7): p. 775-85.

2. Waldock, J., et al., *Assay Harmonization and Use of Biological Standards To Improve the Reproducibility of the Hemagglutination Inhibition Assay: a FLUCOP Collaborative Study.* mSphere, 2021. **6**(4): p. e0056721.

3. Waldock, J., et al., *Haemagglutination inhibition and virus microneutralisation serology assays: use of harmonised protocols and biological standards in seasonal influenza serology testing and their impact on inter-laboratory variation and assay correlation: A FLUCOP collaborative study.* Frontiers in Immunology, 2023. **14**.

4. Peck, H., et al., *Enhanced isolation of influenza viruses in qualified cells improves the probability of well-matched vaccines.* NPJ Vaccines, 2021. **6**(1): p. 149.

5. Oh, D.Y., et al., *MDCK-SIAT1 cells show improved isolation rates for recent human influenza viruses compared to conventional MDCK cells.* J Clin Microbiol, 2008. **46**(7): p. 2189-94.

6. Lin, Y.P., et al., *Evolution of the receptor binding properties of the influenza A(H3N2) hemagglutinin.* Proc Natl Acad Sci U S A, 2012. **109**(52): p. 21474-9.

7. Bernard, M.C., et al., *Validation of a Harmonized Enzyme-Linked-Lectin-Assay (ELLA-NI) Based Neuraminidase Inhibition Assay Standard Operating Procedure (SOP) for Quantification of N1 Influenza Antibodies and the Use of a Calibrator to Improve the Reproducibility of the ELLA-NI With Reverse Genetics Viral and Recombinant Neuraminidase Antigens: A FLUCOP Collaborative Study.* Front Immunol, 2022. **13**: p. 909297.

8. Hobson, D., et al., *The role of serum haemagglutination-inhibiting antibody in protection against challenge infection with influenza A2 and B viruses.* J Hyg (Lond), 1972. **70**(4): p. 767-77.

9. Sicca, F., et al., *Comparison of influenza-specific neutralizing antibody titers determined using different assay readouts and hemagglutination inhibition titers: good correlation but poor agreement.* Vaccine, 2020. **38**(11): p. 2527-2541.

10. Trombetta, C.M., et al., *Comparison of hemagglutination inhibition, single radial hemolysis, virus neutralization assays, and ELISA to detect antibody levels against seasonal influenza viruses.* Influenza and other respiratory viruses, 2018. **12**(6): p. 675-686.

11. Wood, J.M., et al., *Reproducibility of serology assays for pandemic influenza H1N1: Collaborative study to evaluate a candidate WHO International Standard.* Vaccine, 2012. **30**(2): p. 210-217.

12. Vasilijevic, J., et al., *Reduced accumulation of defective viral genomes contributes to severe outcome in influenza virus infected patients.* PLoS Pathog, 2017. **13**(10): p. e1006650.

13. Scull, M.A., et al., *Avian Influenza virus glycoproteins restrict virus replication and spread through human airway epithelium at temperatures of the proximal airways.* PLoS Pathog, 2009. **5**(5): p. e1000424.

14. Matrosovich, M.N., et al., *Human and avian influenza viruses target different cell types in cultures of human airway epithelium.* Proc Natl Acad Sci U S A, 2004. **101**(13): p. 4620-4.

Agenda

Day 1, Wednesday 24 November 2021

[Click here to join the meeting](https://teams.microsoft.com/l/meetup-join/19%3ameeting_ZWQ2ZTc3ODItNzgyZS00ZmI1LWE1NzAtODFkYjM5YTJiOGE5%40thread.v2/0?context=%7b%22Tid%22%3a%22e527ea5c-6258-4cd2-a27f-8bd237ec4c26%22%2c%22Oid%22%3a%2295cb6624-d3e1-4861-b9ec-2bd256ed7a11%22%7d)

| Time UK/CET |  | Topic | Speaker |
| --- | --- | --- | --- |
| 08:00-08:10  9:00-09:10 |  | Introduction Inno4vac | Meta Roestenberg  *Leiden University Medical Center* |
| 08:10-08:30  09:10-09:30 |  | Session 1 Influenza Strain Selection  Need for new Human influenza challenge strain | Christopher Chiu  *Imperial College London* |
| 08:30-09:00  09:30-10:00 |  | Requirements for Human influenza challenge strain | Adrian Wildfire  *CHIMunomics, London* |
| 09:00-09:30  10:00-  10:30 |  | WHO global strategy initiatives and the need for human challenge | Kanta Subbarao  *WHO CC Melbourne* |
| 09:30-10:00  10:30-  11:00 |  | WHO Collaborative Center perspective | John McCauley  *World Influenza Center, Francis Crick Institute London* |
| 10:00-10:30  11:00-11:30 |  | Break |  |
| 10:30-11.00  11:30-12:00 |  | How to choose a new challenge strain using antigenic cartography | Derek Smith  *University of Cambridge* |
| 11:00-11:30  12:00-12:30 |  | Human challenge in context of using in vitro and in vivo models | Wendy Barclay  *Imperial College London* |
| 11:30-12:30  12:30-13:30 |  | Lunch |  |
| 12:30-13:00  13:30-14:00 |  | What drives virus evolution and impact on human challenge strains? | Colin Russell  *University of Amsterdam* |
| 13:00-13:30  14:00-14:30 |  | Human challenge strains and needs for universal vaccine development | Florian Krammer  *Icahn School of Medicine, Mount Sinai, USA* |
| 13:00-14:30  14:30-15:30 |  | Lead panel discussion | Ab Osterhaus  *Tierärtzliche Hochschule, Hannover* |
| 14:30-15:00  15:30-16:00 |  | Break |  |
| 15:00-15:30  16:00-16:30 |  | Session 2 RSV Strain Selection  Strain selection considerations in development of the RSV Maryland challenge agent | Ulla Buchholz  *National Institute of Health, USA* |
| 15:30-16:00  16:30-17:00 |  | Global considerations for RSV challenge strain selection | Charles Sande  *The KEMRI Wellcome Trust Research Programme, Malawi* |
| 16:00-16:30  17:00-17:30 |  | RSV seed viruses under development at Viroclinics | Rienk Jeeninga  *Viroclinics* |
| 16:30-16:45  17:30-17:45 |  | RSV challenge agent strain selection criteria | Ab Osterhaus  *Tierärtzliche Hochschule, Hannover* |
| 16:45-17:30  17:45-18:30 |  | Discussion to finalise RSV strain selection criteria | Christopher Chiu  *Imperial College London* |

Day 2, Thursday 25 November 2021

[Click here to join the meeting](https://teams.microsoft.com/l/meetup-join/19%3ameeting_ZWIxZDM1MjktODAyOC00YjdiLWJlMzgtODUxYTYzN2JiMWIy%40thread.v2/0?context=%7b%22Tid%22%3a%22e527ea5c-6258-4cd2-a27f-8bd237ec4c26%22%2c%22Oid%22%3a%2295cb6624-d3e1-4861-b9ec-2bd256ed7a11%22%7d)

| Time UK/CET |  | Topic | Speaker |
| --- | --- | --- | --- |
| 09:00-10:00  10:00-11:00 |  | Session 3 Internal discussion WP8 Influenza strain selection  *Closed session*  Strain selection flu and way forward (Recap 24^th^) | Rebecca Cox, Christopher Chiu, Othmar Engelhardt, Fabienne Piras, Nathalie Mantel, Anke Huckriede, Puck Van Kasteren, Fan Zhou |
| 10:00-10:30  11:00  11:30 |  | Break |  |
| 10:30-10:45  11:30-11:45 |  | Session 4 Influenza Immunological Assays  Pre-existing immunity and correlates of protection | Rebecca Cox  *University of Bergen* |
| 10:45-11:10  11:45-  12:10 |  | Serological assays standardised in Flucop | Joanna Waldock  *MHRA* |
| 11:10-11:25  12:10-  12:25 |  | Harmonising stalk assays | Othmar Engelhardt  *MHRA* |
| 11:15-11:40  12:25-12:40 |  | Discussion |  |
| 11:40-11:50  12:40-12:50 |  | Break |  |
| 11:50-12:20  12:50-13:20 |  | Measuring local IgA | John Tregoning & Ryan Thwaites  *Imperial College London* |
| 12:20-13:00  13:20-14:00 |  | Discussion | Katja Hoschler  *UKHSA* |
| 13:00-13:15  14:00-14:15 |  | In vitro models | Puck Van Kasteren& Anke Huckriede  *RVIM& UNISI* |
| 13:15-14:15  14:15-15:15 |  | Lunch |  |
| 14:15-14:40  15:15-15:40 |  | Session 5 Immunological Assays  RSV standardisation neutralisation assays | Jacqueline McDonald  *MHRA* |
| 14:40  15:10  15:40-16:10 |  | Antibody competition assays | Steff Ascough  *Imperial College London* |
| 15:10  15:30  16:10-16:30 |  | T cells | Guus Rimmelzwaan  *Tierärtzliche Hochschule, Hannover* |
| 15:30-  16:15  16:30-17:15 |  | Way forward for RSV assays and harmonisation of assays | Chair: Maria Zambon & Othmar Engelhardt  *UKHSA & MHRA* |
| 16:15  17: 15 |  | End of Meeting |  |
